# Antivirals Reduce the Formation of Key Alzheimer's Disease Molecules in Cell Cultures Acutely Infected with Herpes Simplex Virus Type 1

**DOI:** 10.1371/journal.pone.0025152

**Published:** 2011-10-07

**Authors:** Matthew A. Wozniak, Alison L. Frost, Chris M. Preston, Ruth F. Itzhaki

**Affiliations:** 1 Faculty of Life Sciences, The University of Manchester, Manchester, United Kingdom; 2 Centre for Virus Research, Medical Research Council-University of Glasgow, Glasgow, United Kingdom; University of Cambridge, United Kingdom

## Abstract

Alzheimer's disease (AD) afflicts around 20 million people worldwide and so there is an urgent need for effective treatment. Our research showing that herpes simplex virus type 1 (HSV1) is a risk factor for AD for the brains of people who possess a specific genetic factor and that the virus causes accumulation of key AD proteins (β-amyloid (Aβ) and abnormally phosphorylated tau (P-tau)), suggests that anti-HSV1 antiviral agents might slow AD progression. However, currently available antiviral agents target HSV1 DNA replication and so might be successful in AD only if Aβ and P-tau accumulation depend on viral DNA replication. Therefore, we investigated firstly the stage(s) of the virus replication cycle required for Aβ and P-tau accumulation, and secondly whether antiviral agents prevent these changes using recombinant strains of HSV1 that progress only partly through the replication cycle and antiviral agents that inhibit HSV1 DNA replication. By quantitative immunocytochemistry we demonstrated that entry, fusion and uncoating of HSV1, are insufficient to induce Aβ and P-tau production. We showed also that none of the “immediate early” viral proteins is directly responsible, and that Aβ and P-tau are produced at a subsequent stage of the HSV1 replication cycle. Importantly, the anti-HSV1 antiviral agents acyclovir, penciclovir and foscarnet reduced Aβ and P-tau accumulation, as well as HSV1, with foscarnet being less effective in each case. P-tau accumulation was found to depend on HSV1 DNA replication, whereas Aβ accumulation was not. The antiviral-induced decrease in Aβ is attributable to the reduced number of new viruses, and hence the reduction in viral spread. Since antiviral agents reduce greatly Aβ and P-tau accumulation in HSV1-infected cells, they would be suitable for treating AD with great advantage unlike current AD therapies, only the virus, not the host cell, would be targeted.

## Introduction

Alzheimer's disease (AD) is a prevalent neuropsychiatric disorder that primarily affects the elderly. It is characterised by memory loss and cognitive dysfunction, and examination of sufferers' brains reveals an abundance of two neuropathological features – senile plaques and neurofibrillary tangles. Plaques and tangles, and their components – β-amyloid (Aβ) and abnormally phosphorylated tau (P-tau), respectively – are thought to be central to disease pathogenesis but their cause and thus the underlying cause of AD is unknown.

Current treatments for AD are merely palliative and thus there is an urgent need for medications that delay disease progression. One possible treatment strategy is the use of antiviral agents, in particular agents that are effective against herpes simplex virus type 1 (HSV1). This suggestion is based on the increasing body of evidence implicating HSV1 in the aetiology of AD.

HSV1 is a neurotropic virus that infects most humans. It is responsible for a number of diseases including herpes labialis (cold sores), herpes simplex encephalitis (HSE) and some cases of genital herpes. The first suggestion that HSV1 might have a role in AD was based on the observation that in HSE the brain regions damaged are the same ones as those that are affected in AD [1]. Subsequent research demonstrated that the virus is present in the brain of most elderly subjects, including AD sufferers, and that it is present within the brain regions affected by AD [2]. Moreover, the virus was found to be a risk factor for the disease when in brain of possessors of a specific genetic factor – the type 4 allele of the apolipoprotein E gene [3]–[5]. Additional studies have linked HSV1 directly to the neuropathological features of AD. Specifically, HSV1 infection of cells in culture causes Aβ and P-tau accumulation [6]–[12] and the brains of HSV1-infected mice exhibit Aβ deposits [7]. Consistently, the enzymes involved in the generation of Aβ and of (P-tau are increased in HSV1-infected cells [6], [7], [13]. Further, the distribution of HSV1 DNA in human brains in relation to senile plaques has been investigated using a combination of *in situ* polymerase chain reaction (which amplifies and thereby detects low levels of target DNA sequences in tissues) with immunohistochemistry for Aβ, or thioflavin S staining for plaques. This revealed that HSV1 DNA is located very specifically within senile plaques in AD frontal and temporal cortex [14]. The *in situ* polymerase chain reaction findings do not prove HSV1-AD causality, but taken with the increased Aβ caused by infection, they suggest that HSV1 is involved in formation of plaques (and toxic Aβ oligomers), and hence support a causal role for in AD.

As mentioned above, the role of HSV1 in AD points to the use of antiviral agents to slow the progression of the disease. However, currently available antiviral agents target viral DNA replication and so might only be effective in AD if the accumulation of Aβ and P-tau depends on viral DNA replication. If, on the other hand, Aβ and P-tau are produced independently of viral DNA replication then current antiviral agents might not completely inhibit the production of these proteins, and thus might have a limited effect on disease progression. The aim of the present study was to determine the stage of the virus replication cycle necessary for the accumulation of Aβ and P-tau and to determine whether antiviral agents do reduce the accumulation of Aβ and P-tau. Using HSV1 recombinants, we show that Aβ and P-tau accumulation requires the initiation of early and/or late protein synthesis. We found also that antiviral agents indeed reduce the accumulation of Aβ and P-tau in HSV1-infected cell cultures – supporting the usage of antiviral agents to treat AD.

## Results

### Aβ and P-tau accumulation requires early and/or late HSV1 protein synthesis

The infectious cycle of HSV1 is a complex process, which begins with binding of the virus to heparan sulfate proteoglycan molecules on the cell surface. Subsequently the virus binds to more specific receptors which facilitate virus entry into the cell by fusion [15]. Once inside the cell the virus uncoats, the viral DNA moves to the nucleus and circularises, and virus gene expression occurs. Gene expression is initiated by a protein complex consisting of a viral protein (virion protein (VP16)) and two cellular proteins (host cell factor 1 and octomer-binding protein 1 (oct1)) [16]. There are three main classes of genes which are expressed at different times during infection, and are termed immediate early (IE), early and late (or alpha, beta and gamma) [17]. IE proteins include infected cell polypeptide (ICP) 0 and ICP4 which are transcriptional activators [18], [19]. Early proteins include those proteins required for viral DNA replication such as the protein expressed from the unique long 42 (UL42) gene [20]. Late proteins include components of the virus particle such as glycoprotein C (gC) and proteins required for virus assembly and egress. Expression of gC is strictly dependent upon viral DNA synthesis [21]. Once sufficient levels of virion proteins accumulate, virus particles assemble and the virus exits the cell. Aβ and P-tau accumulation might require any of the stages or components of the infectious cycle of HSV1.

**Figure 1:**
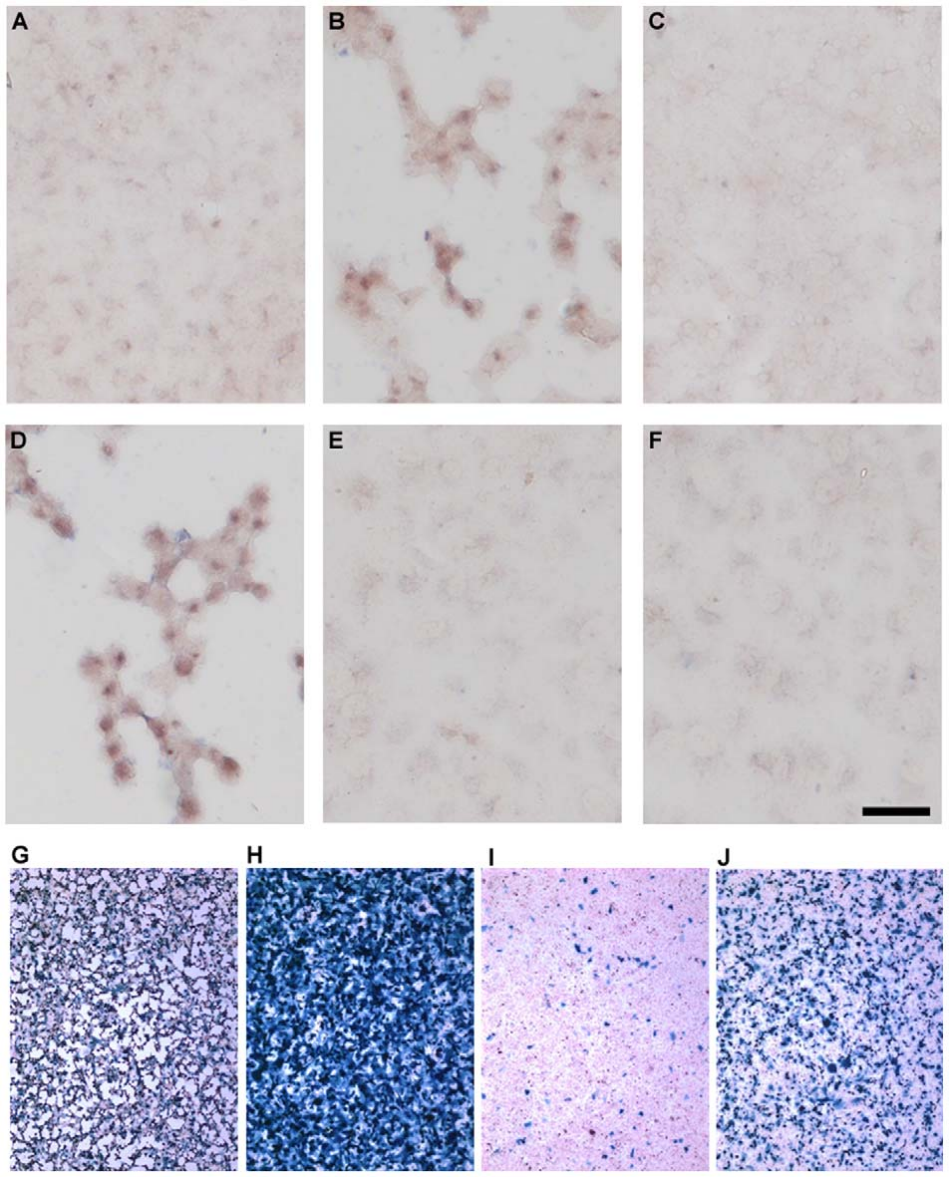
Initiation of Aβ accumulation requires the initiation of early and/or late protein synthesis. Vero cells were infected for 16 hours with HSV1 strain 17 or with HSV1 recombinants, each at an MOI of 5, and tested for Aβ by immunocytochemistry. Infection with *in*1374 did not lead to Aβ accumulation (A) whereas infection with wild type HSV-1 (*in*1863) did show Aβ accumulation (B). Infection with recombinant *ts*K*/lacZ* showed no Aβ accumulation at the restrictive temperature (C). Infection with recombinant *in*1404 did show Aβ staining (D). Mock-infected cells at 37°C (E) or at 38.5°C (F) showed no staining. Scale bar: 50 µm. To confirm infection of cells, monolayers were stained for the expression of β-gal. Infection with *in*1863 (G) or *ts*K/*lacZ* (H) resulted in β-gal expression in most cells. In cultures infected with *in*1374 only a small proportion of cells expressed β-gal (I), whereas many more were positive after co-infection with *in*1374 and WT HSV1 (MOI 5 for each virus), demonstrating that *in*1374 had entered a high proportion of cells (J). Cells show typical HSV1-cytopathic effects, i.e., they become rounded and contracted.

**Figure 2:**
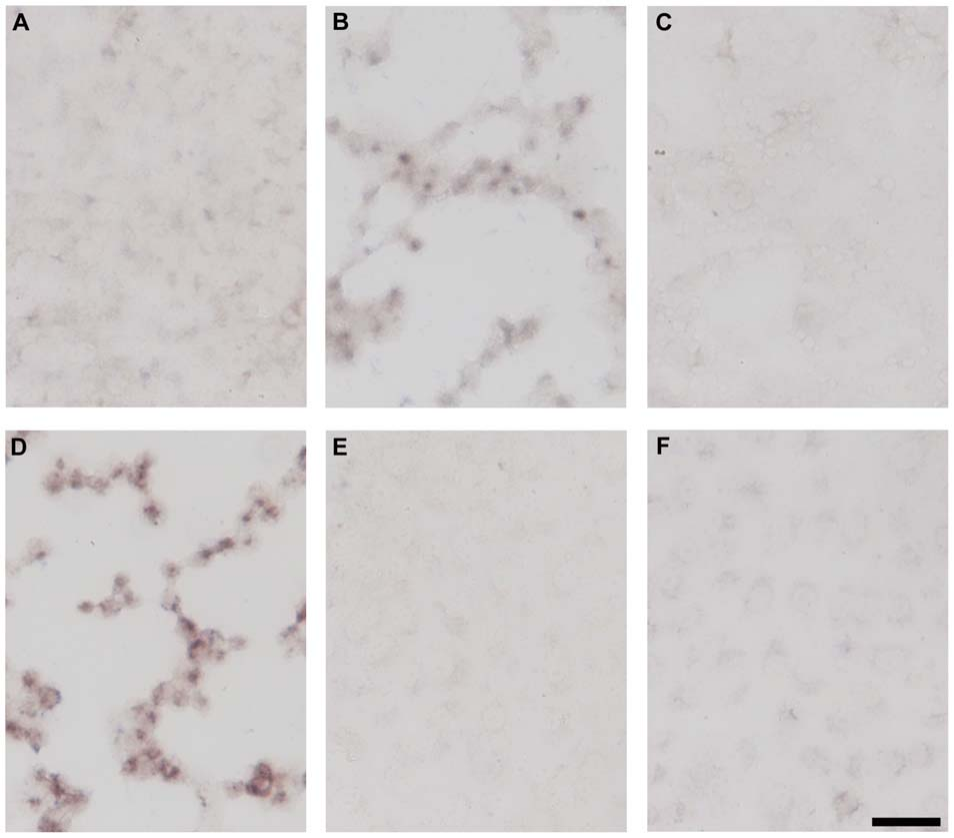
Initiation of abnormal tau phosphorylation requires the initiation of early and/or late protein synthesis. Vero cells were infected for 16 hours with HSV1 strain 17 or with HSV1 recombinants, each at an MOI of 5, and tested for abnormal tau phosphorylation (pS214) by immunocytochemistry. Infection with *in*1374 did not lead to abnormal tau phosphorylation (A) whereas infection with *in*1863 did show phosphorylation (B). Infection with recombinant *ts*K/*lacZ* showed no abnormal tau phosphorylation at the restrictive temperature (C). Infection with recombinant *in*1404 did show staining (D). Mock-infected cells at 37°C (E) or at 38.5°C (F) showed no staining. Scale bar: 50 µm.

In order to find which stage of the infection cycle is involved in the production of Aβ and P-tau, we used HSV-1 recombinants blocked at early stages in the infection cycle. *in*1374 is a multiple recombinant that enters cells and is uncoated but does not synthesise any HSV1 proteins to detectable levels in most infected cells [22]. Infection of Vero cells with this recombinant, at the restrictive temperature, did not result in accumulation of Aβ or P-tau (Figure 1A and 2A), whereas infection with the wild-type (WT) virus *in*1863 did cause Aβ and P-tau accumulation (Figure 1B and 2B). Recombinant *tsK/lacZ*, a virus with a temperature-sensitive (*ts*) mutation in ICP4, at the restrictive temperature expresses IE proteins at high levels but does not synthesize subsequent early or late proteins [23]. This recombinant did not show Aβ or P-tau accumulation (Figure 1C and 2C). These results suggest that neither binding and entry of HSV1 nor the production of IE proteins is sufficient to cause accumulation of Aβ and P-tau. Their accumulation therefore requires the production of early and/or late HSV-1-specified proteins.

A viable HSV1 recombinant that specifies a truncated glycoprotein E (gE) (*in*1404), and fails to assemble a functional Fc receptor [24], did produce Aβ and P-tau accumulation (Figure 1D and 2D), suggesting that the Aβ and P-tau staining we observe is not an artefact due to the binding of the primary or secondary antibodies to the gE-glycoprotein I Fc receptor encoded by HSV1.

The efficiency of infection with *in*1863, *ts*K/*lacZ* and *in*1374 was investigated by staining monolayers for the presence of β-gal. In cultures infected with *in*1863 or *ts*K/*lacZ*, most cells were positive for the enzyme (Figure 1 G and H). This demonstrates efficient primary infection at MOI 5, since *ts*K/*lacZ* does not replicate at the nonpermissive temperature of 38.5°C. Few cells expressed detectable levels of β-gal in *in*1374-infected cultures (Figure 1 I). However, many more were positive for β-gal after co-infection with WT HSV1 lacking a *lacZ* insertion (Figure 1 J), because co-infection provided functional VP16, ICP0 and ICP4.

### Inhibition of HSV1 DNA replication reduces the accumulation of Aβ and P-tau in HSV1-infected cells

We investigated whether HSV1 DNA replication was necessary for the accumulation of Aβ and P-tau by using the antiviral agent acyclovir (ACV), which inhibits HSV1 DNA replication. ACV targets specifically cells that contain replicating HSV1 in that its action requires phosphorylation by the viral thymidine kinase (TK), a more effective phosphorylating agent than cellular purine or pyrimidine kinases. The monophosphate form of ACV is then further phosphorylated by cellular kinases to the active triphosphate form, which has a greater affinity for viral than for cellular DNA polymerase. Acyclo-GTP is incorporated into viral DNA, causing chain termination; thus virus replication and spread are aborted and consequent tissue damage is halted.

**Figure 3:**
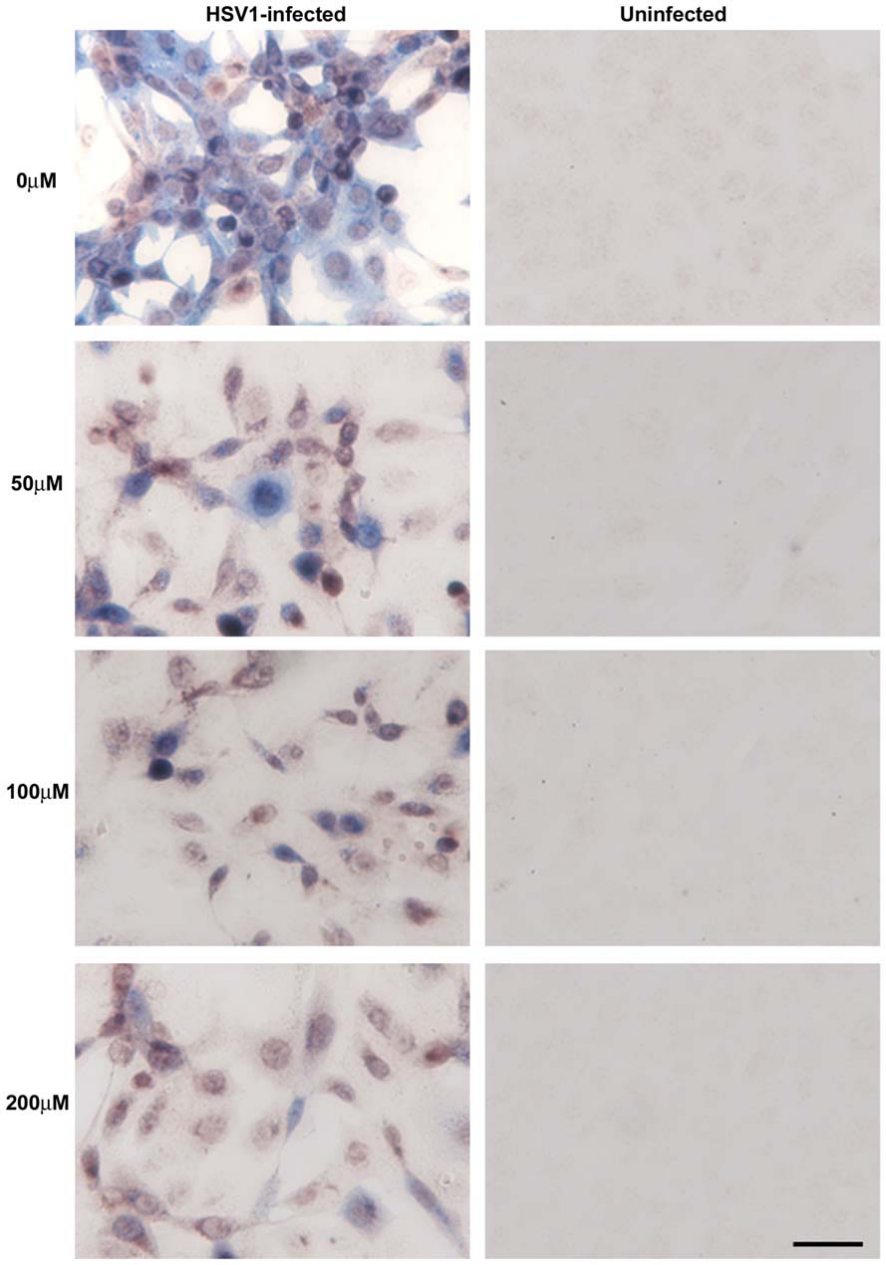
Acyclovir inhibits HSV1 replication. Vero cells were infected with HSV1 SC16 at an MOI of 1 for 16 hours. Cells were treated with 0 µM, 50 µM, 100 µM or 200 µM acyclovir (ACV), which was present throughout infection. After fixation the slides were tested for HSV1 proteins using immunocytochemistry. Reactivity with HSV1 proteins was significantly reduced in the presence of ACV. Scale bar: 50 µm.

**Figure 4:**
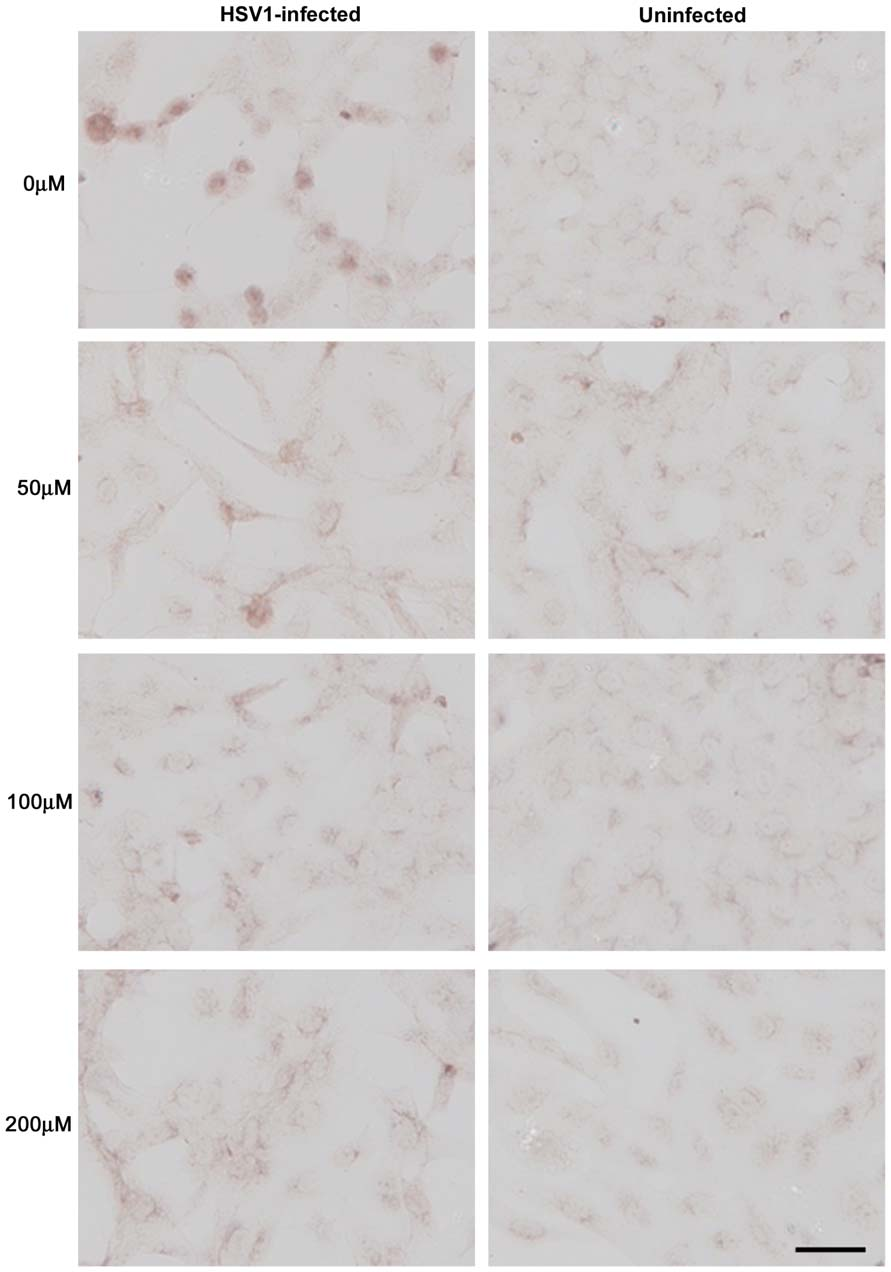
Acyclovir inhibits HSV1-induced β-amyloid accumulation. Vero cells were infected with HSV1 SC16 at an MOI of 1 for 16 hours. Cells were treated with 0 µM, 50 µM, 100 µM or 200 µM acyclovir (ACV), which was present throughout infection. After fixation the slides were tested for β-amyloid (Aβ) accumulation using immunocytochemistry. These results clearly show that ACV reduces Aβ significantly. Scale bar: 50 µm.

**Figure 5:**
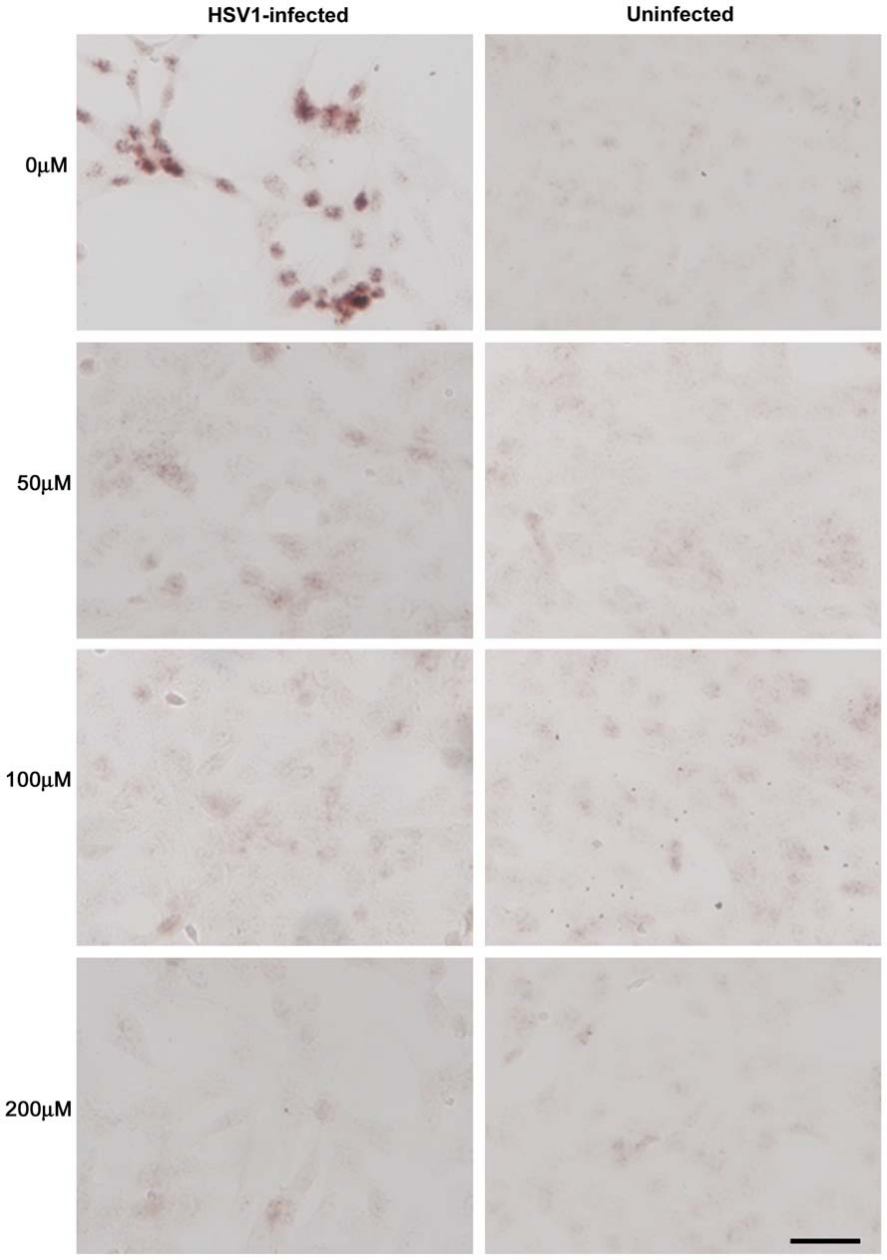
Acyclovir inhibits HSV1-induced abnormal tau phosphorylation. Vero cells were infected with HSV1 SC16 at an MOI of 1 for 16 hours. Cells were treated with 0 µM, 50 µM, 100 µM or 200 µM acyclovir (ACV), which was present throughout infection. After fixation the slides were tested for abnormal tau phosphorylation (AT100) using immunocytochemistry. ACV significantly reduces AT100 staining. Scale bar: 50 µm.

**Figure 6:**
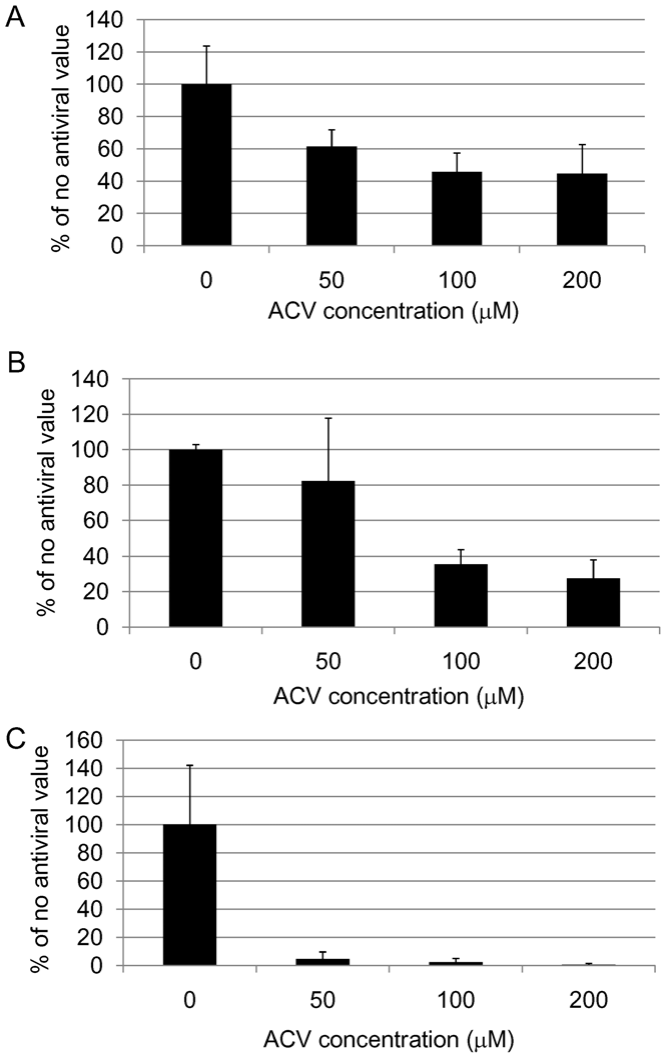
Quantification of HSV1 proteins, β-amyloid and abnormal tau phosphorylation in HSV1-infected cells after acyclovir treatment. Vero cells were infected with HSV1 SC16 at an MOI of 1 for 16 hours. Cells were treated with 0 µM, 50 µM, 100 µM or 200 µM acyclovir (ACV), which was present throughout infection. After fixation the slides were tested for HSV1 proteins, β-amyloid (Aβ) accumulation and abnormal tau phosphorylation (AT100) using immunocytochemistry and the amount of staining was quantified using Image J. Values are expressed as a percentage of staining produced when no antiviral is used. For HSV1 proteins (A) and abnormal tau phosphorylation (C), there is a statistically significant decrease in staining for all concentrations of ACV tested compared to the staining in cells infected but not treated with ACV (p<0.0001 in both cases). For Aβ (B), there is a statistically significant decrease in staining with 100 µM ACV and 200 µM only (p<0.0001). ANOVA was used to test for significance. The results are a combination of two independent experiments.

We infected Vero cells with HSV1 at a multiplicity of infection (MOI) of 1 and examined the effects of varying doses of ACV on HSV1, Aβ and P-tau by immunocytochemistry (ICC). Figures 3, 4 and 5 clearly show a reduction in amounts of HSV1 proteins, Aβ and P-tau in the presence of ACV. Also, at 200 µM ACV the cells exhibit far less cytopathology. Quantification of these ICC results using Image J reveals that there is a significant reduction in HSV1 proteins and abnormal tau phosphorylation at all concentrations used (p<0.0001 in both cases) and in Aβ at 100 µM and 200 µM (p<0.0001) (Figure 6). The staining for P-tau dropped to almost zero at 50 µM, but that of Aβ decreased less: at 200 µM ACV its level was 28% of the infected cell value in the absence of antiviral, significantly different (p<0.05) from the value for mock-infected cells, which corresponded to about 10% of the infected cell value.

Similarly, ACV treatment did not cause HSV1 staining to disappear completely. This might reflect the possibility that the HSV1 strain we used was ACV-resistant, or that the staining was caused by proteins produced independently of viral DNA replication. Therefore the reduction of Aβ to only 28% might be related also to the use of an ACV-resistant strain or to the possibility that it is produced independently of viral DNA replication. We checked that the strain of virus we were using was not resistant to some extent to the antiviral agent using plaque reduction assay (PRA) to determine the dose of ACV which reduces infectivity to 50% (IC50). In this method, 24-well plates were seeded with Vero cells, HSV1 was added and the spread of the virus was restricted to two dimensions by the use of a dense medium which results in the formation of plaques (holes within the monolayer of cells). The dose of HSV1 used was chosen in order to produce a number of plaques that is easy to count (usually around 80 plaques per well). Addition of an antiviral agent reduces the number of plaques per well and, if several doses of antiviral are used, the IC50 can be determined. Using PRA we found that the IC50 value of ACV was 0.83 µM, which is consistent with published values [25], and suggests that the strain of HSV1 we used was not resistant to ACV. An alternative explanation for why HSV1 staining did not disappear completely could be because the antibody we used detected multiple HSV1 proteins. Thus the staining detected might represent proteins that are expressed independently of viral DNA replication. Therefore we looked at the effects of ACV on specific HSV1 proteins using monoclonal antibodies specific for ICP0, UL42 and gC, which represent proteins from the immediate early, early and late stages, respectively, of the virus infectious cycle. We found that staining for gC (a late protein that is dependent on DNA replication) was reduced to less than 1% of the value in the absence of antiviral, whereas staining for ICP0 (an IE protein) and UL42 (an early protein), which are independent of viral DNA replication [17], was reduced but not to the same extent (to ∼20% in each case – data not shown). This shows that the HSV1 staining occurring even at high ACV doses presumably reflects these and other proteins that are synthesised independently of viral replication, and suggests that Aβ accumulation too is independent of viral replication.

### Other antiviral agents produce effects similar to those of ACV

To discover whether other antiviral agents affect Aβ and P-tau accumulation, we investigated penciclovir (PCV) and foscarnet (FOS). PCV, another guanine analogue, like ACV depends on the presence of the HSV1 TK, and its products block chain elongation of the viral DNA, but the two agents differ in their affinity for TK and the viral DNA polymerase: that of ACV is much greater for the TK, and that of PCV (as triphosphate) is much greater for the polymerase [26]. FOS acts by inhibiting the pyrophosphate binding site on viral DNA polymerases, at levels that do not affect human DNA polymerases [27].

**Figure 7:**
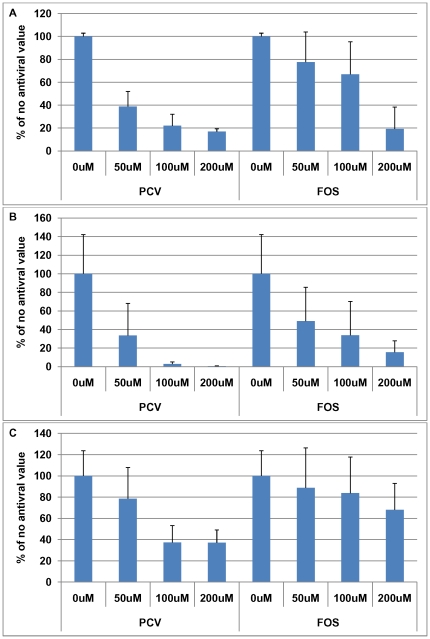
Comparison of different antiviral agents on proteins accumulating during HSV1 infection. Vero cells were infected with HSV1 SC16 at an MOI of 1 for 16 hours. Cells were treated with 0 µM, 50 µM, 100 µM or 200 µM penciclovir (PCV) or foscarnet (FOS) which was present throughout infection. After fixation the slides were tested for (A) β-amyloid (Aβ) accumulation, (B) abnormal tau phosphorylation (AT100), and (C) HSV1 proteins using immunocytochemistry and the amount of staining was quantified using Image J. Values are expressed as a percentage of staining produced when no antiviral is used. These values were used to determine the antiviral concentration required to reduce staining to 50%. The results are a combination of two independent experiments. Values for mock-infected cells were between 0 and 8% (data not shown).

Both PCV and FOS reduced accumulation of Aβ (Figure 7A). The decreases in Aβ level were statistically significant at all concentrations of PCV used (p<0.0001) whereas FOS reduced Aβ levels significantly only at 200 µM (p<0.0001). Comparison of the different antiviral agents reveals that for Aβ levels, at a concentration of 50 µM the reduction caused by PCV was significantly different from those of the other antiviral agents (p = 0.025); at 100 µM, the reductions caused by PCV and ACV were significantly different from that of FOS (p = 0.003), but at 200 µM, there were no significant differences amongst the antiviral agents. Consistently, the concentration of antiviral agent required to reduce the ICC signal of Aβ to 50% was lowest for PCV (40.8 µM), followed by ACV (84.4 µM) and then FOS (135.3 µM).

PCV and FOS reduced P-tau level too. The decreases in P-tau level were statistically significant at all concentrations of PCV used (p<0.0001) whereas FOS reduced P-tau levels significantly at 100 µM and 200 µM (p = 0.012). However, there were no significant differences in P-tau staining amongst the antiviral agents at each concentration. Consistently, the concentrations of antiviral agents required to reduce the ICC signal of P-tau to 50% were fairly similar for ACV (26.1 µM), PCV (37.6 µM) and FOS (48.9 µM).

HSV1 levels were statistically significantly reduced at 100 µM and 200 µM PCV (p<0.0001) but not at any concentration of FOS (p = 0.182). The difference in HSV1 protein staining amongst the antiviral agents at 50 µM was not significant (p = 0.139). At 100 µM and 200 µM the differences were statistically significant (p = 0.001 and p = 0.005, respectively), with PCV and ACV causing a greater reduction than FOS. Consistently, the concentrations of ACV and PCV required to reduce the ICC signal of HSV1 proteins to 50% were similar (86.1 µM and 84.5 µM, respectively) and FOS was lower (>200 µM).

These results show that FOS is less effective than ACV or PCV not only at reducing HSV1 replication but also at reducing Aβ and P-tau production.

As with ACV, PCV reduced P-tau staining to levels similar to those in mock-infected cells (Figure 7B) but Aβ level reached a plateau of ∼20% (Figures 7A), which was statistically significantly higher than the values obtained for mock-infected cells. As with ACV, we used PRA to check that the strain of HSV1 was not resistant to PCV. We found that the IC50 for PCV was 7.2 µM, indicating that the strain was not PCV-resistant. We investigated also the effects of PCV on HSV1 ICP0, UL42 and gC and found similar results (data not shown) to those of ACV, suggesting again that Aβ production is produced independently of HSV1 DNA replication.

**Figure 8:**
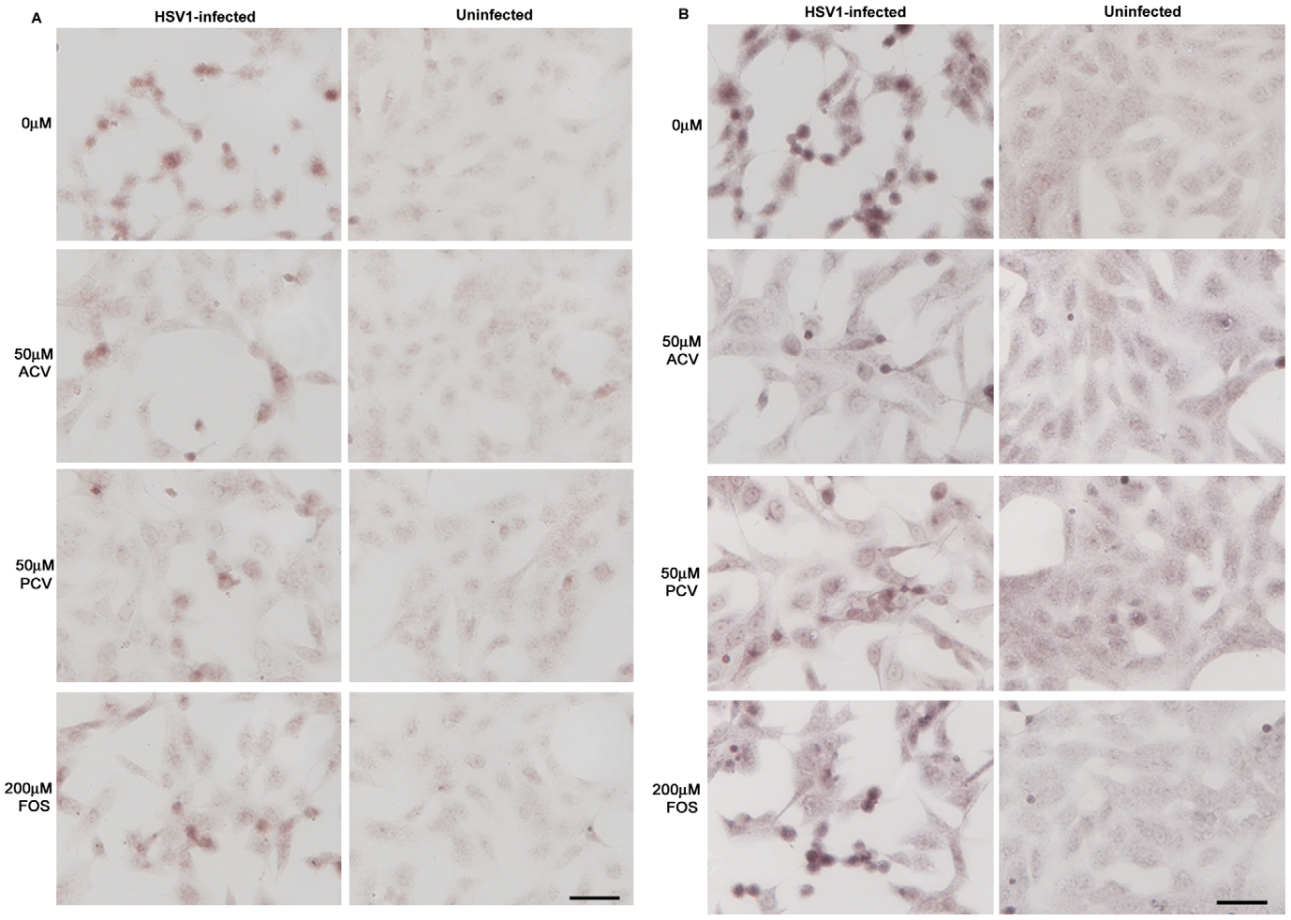
Antiviral agents reduce the HSV1-induced increases in β-amyloid-related enzymes. Vero cells were infected with HSV1 SC16 at an MOI of 1 for 16 hours. Cells were treated with 0 µM, 50 µM, 100 µM or 200 µM acyclovir (ACV), penciclovir (PCV) or foscarnet (FOS) which was present throughout infection. After fixation the slides were tested for (A) β-site amyloid precursor protein cleaving enzyme 1 (BACE1) and (B) nicastrin. Images for no antiviral, 50 µM ACV, 50 µM PCV and 200 µM FOS are shown. Scale bar: 50 µm.

In the case of FOS treatment, P-tau and Aβ were reduced to ∼15% and ∼20%, respectively (Figures 8A and 8B). We examined the effects of FOS on HSV1 gC, UL42 and ICP0. FOS reduced the levels of all these proteins but in all cases the levels did not decrease to the levels found in mock-infected cells (data not shown). This might reflect the use of a FOS-resistant strain of HSV1 (the IC50 in PRA was >100 µM but this is consistent with published values), the poor antiviral ability of FOS and/or DNA replication-independent accumulation.

### Comparison of quantitative immunocytochemistry with HSV1 ELISA

As quantitative ICC (qICC) is not a standard method, we compared the results of the qICC for HSV1 proteins with HSV1 ELISA (PRA could not be used as conditions comparable to qICC (i.e., an MOI of 1, and 16 hours infection would be unsuitable as at this dose, plaque numbers would be vastly too high). HSV1 ELISA revealed that the IC50 values for ACV, PCV and FOS were 81.6 µM, 183 µM and >200 µM, respectively. These ELISA values are in agreement with those obtained by qICC, confirming the validity of the latter.

### Inhibition of HSV1 DNA replication reduces the enzymes involved in accumulation of Aβ and P-tau in HSV1-infected cells

**Figure 9:**
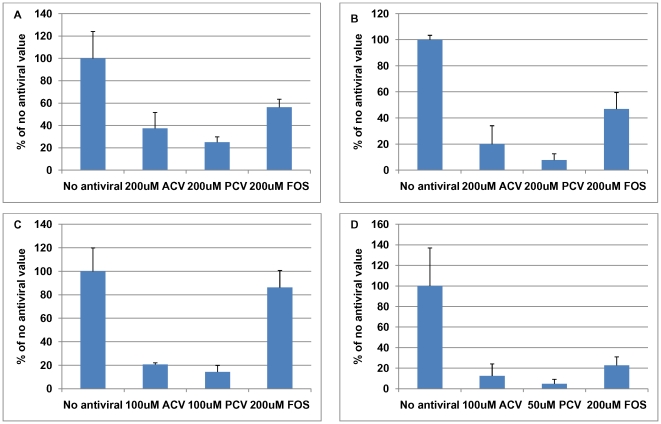
Quantification of the effect of antiviral agents on the HSV1-induced increases in amyloid- and tau-related enzymes. The immunocytochemistry results in Figures 8 and 9 were quantified using Image J. (A) β-site amyloid precursor protein cleaving enzyme 1, (B) nicastrin, (C) protein kinase A and (D) glycogen synthase kinase 3β.

HSV1 infection increases the levels of enzymes (or components of the enzymes) involved in Aβ and P-tau formation [7]. We therefore examined the effects of the antiviral agents on these levels and found that the virus-induced increases in the Aβ-related enzyme, β-site amyloid precursor protein cleaving enzyme 1 (BACE1), and a component of the γ-secretase, nicastrin, were reduced upon treatment with the antiviral agents (Figure 8). Quantification of these results showed that FOS is less effective than ACV and PCV at reducing BACE1 and nicastrin (Figure 9). Consistently, the concentrations of ACV, PCV and FOS required to reduce staining to 50% were 46.9, 35.1 and >200 µM for BACE1 and 54.1, 69.9 and >200 µM for nicastrin. Interestingly, values for BACE1 and nicastrin did not drop to the levels obtained for mock-infected cells (which were 0-10% for both BACE1 and nicastrin).

**Figure 10:**
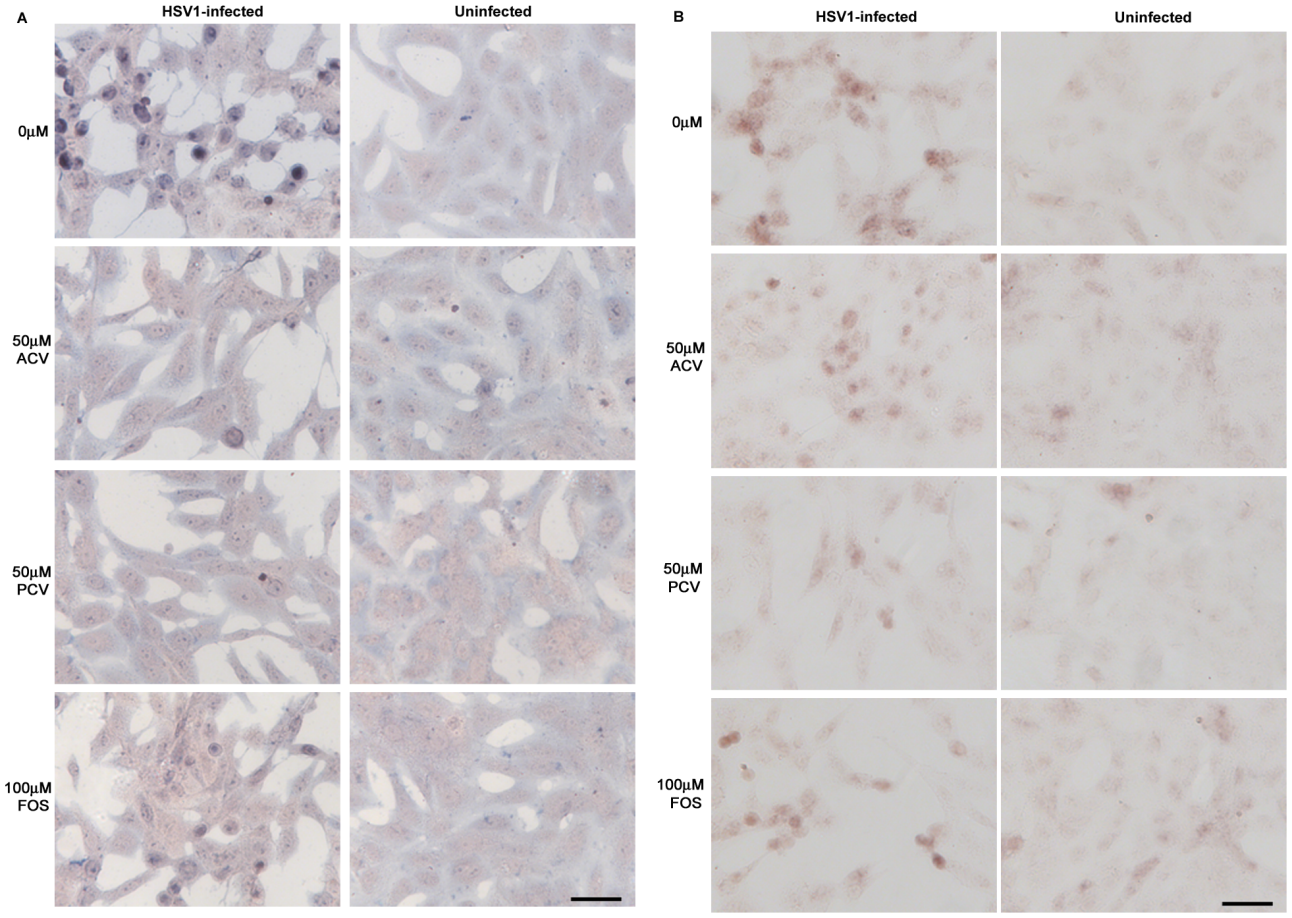
Antiviral agents reduce the HSV1-induced increases in tau-related enzymes. Vero cells were infected with HSV1 SC16 at an MOI of 1 for 16 hours. Cells were treated with 0 µM, 50 µM, 100 µM or 200 µM acyclovir (ACV), penciclovir (PCV) or foscarnet (FOS) which was present throughout infection. After fixation the slides were tested for (A) protein kinase A (PKA) and (B) glycogen synthase kinase 3β (GSK3β). Images for no antiviral, 50 µM ACV, 50 µM PCV and 100 µM FOS are shown. Scale bar: 50 µm.

Similarly the HSV1-induced increases in the P-tau-related enzymes, protein kinase A (PKA) and glycogen synthase kinase 3β (GSK3β), are reduced after treatment with ACV, PCV and FOS (Figure 10). Quantification of the results revealed that FOS is less effective than ACV and PCV at reducing PKA and GSK3β (Figure 9). GSK3β staining dropped to levels found in mock-infected cells (which were 10-20% of the HSV1-infected, no antiviral values) at 100 µM ACV, 50 µM PCV and 200 µM FOS, and PKA staining dropped to the levels found in mock-infected (which were ∼20% of the HSV1-infected, no antiviral values) at 100 µM ACV and 100 µM PCV. The levels of PKA after FOS treatment did decrease, but did not fall to levels for mock-infected cells even at 200 µM. The concentrations of ACV, PCV and FOS required to reduce staining to 50% were 60.7, 47.9 and 136.4 µM for PKA and 33.4, 26.5 and 94.7 µM for GSK3β.

## Discussion

Our main findings are that the three antiviral agents we studied significantly reduced the levels of Aβ and P-tau and inhibited too the replication of HSV1. Our data using the recombinant viruses suggest that initial events in the infectious cycle of HSV1, such as entry, fusion and uncoating, are not sufficient to induce the accumulation of Aβ nor are they sufficient for phosphorylation of tau at pS214. They show also that none of the IE proteins is directly responsible for these effects since the IE proteins accumulate to high levels during infection with the *ts*K recombinant but Aβ and P-tau are not formed, although we cannot rule out the possibility that functional ICP4 proteins are directly needed. Therefore the results with the recombinants suggest that Aβ and P-tau are produced after the IE stage of the replication cycle of HSV1.

Our results with the antiviral agents suggest that P-tau formation (as shown by the AT100 antibody) is dependent on viral DNA replication directly or on a protein that depends on viral DNA replication, as treatment with ACV and PCV reduced the AT100 antibody staining to a very low level (∼1%). Consistently, staining for gC, a viral protein that is known to be dependent on viral DNA replication, was reduced to less than 1% after treatment with ACV and PCV. FOS did not produce a decrease to the same extent as the other two agents but this likely reflects the fact that FOS is not as effective as ACV and PCV at inhibiting viral DNA replication. Consistently, ACV and PCV treatment led to a drop in the staining for the relevant phosphorylating enzymes – PKA and GSK3β – to levels similar to those in mock-infected cells.

In contrast to P-tau, Aβ accumulation was not completely inhibited by antiviral treatment; even at a high dose (200 µM) there was still accumulation which was around 20–30% (depending on the agent used) of the amount in the absence of antiviral, a value statistically significantly different from those for mock-infected cells. Although our results do not preclude higher doses preventing Aβ accumulation completely, the fact that the staining level of Aβ is beginning to plateau with both ACV and PCV, and that staining of gC and of P-tau was reduced by the antiviral agents to less than 1%, suggests that Aβ is indeed produced independently of viral DNA replication. Accumulation of Aâ was inhibited by ACV to approximately the same extent (70–80%) as production of the IE protein ICP0 and the early protein UL42. Synthesis of HSV1-specified IE and early proteins is not affected by inhibition of viral DNA synthesis, therefore we can infer that about 20–30% of cells were primarily infected and that the ACV-mediated reduction in ICP0, UL42 and Aâ was due to prevention of subsequent virus production and spread. We can infer also that Aβ accumulation cannot be a result of virus binding to the cell surface or of its entry, as it was not caused by the *in*1374 recombinant virus, which binds to and enters cells but does not progress further. Further, Aβ accumulation cannot be a direct effect of the IE proteins as it was not produced by infection with the *ts*K/*lacZ* recombinant at the restrictive temperature, which synthesises IE proteins but progresses no further. Instead it must be due to an early and/or a late protein (one not dependent on DNA replication). Consistent with the Aβ staining, the staining of the Aβ-related enzymes, BACE1 and nicastrin, did not decrease to the levels found in mock-infected cells, suggesting that they too are induced independently of viral DNA replication; however, we did not investigate their levels with the recombinant viruses, so we cannot deduce what stage or protein(s) is responsible for their increase.

Two other groups have recently investigated HSV1-induced Aβ accumulation. Firstly, Piacentini et al. [9] showed that Aβ accumulation occurs in HSV1-infected primary cultures of rat embryonic neurons. They used also UV-inactivated HSV1 (which binds to and enters cells but does not replicate) and found that it did not produce Aβ. This is consistent with our results with the recombinant strains of HSV1. Secondly, Santana et al. [10] found that in HSV1-infected amyloid precursor protein-transfected human neuroblastoma cells, Aβ accumulation occurred, consistent with our data, but they detected it two hours post infection, well before the start of viral DNA replication. Our non-detection of Aβ production at such an early time might have been due to our using a much lower HSV1 dose – 1 plaque-forming unit (pfu)/cell – than their 10 pfu/cell, and/or to a cell type difference: we used non-APP-transfected Vero cells. Another difference is our finding that FOS decreased Aβ levels whereas in their study it did not visibly affect Aβ staining, even at the high concentration of 1.3 mM, although it presumably reduced or stopped viral replication and spread during the 18 hour infection. This too might be related to their higher viral dose: theirs would have infected 100% of cells compared to our 20–30%, thus producing far more Aβ, so that neither FOS, a relatively inefficient inhibitor of Aβ, nor the subsequent stoppage of viral replication would have had an appreciable effect on Aβ level.

Two other studies have investigated the effect of an antiviral on processes relating to those occurring in AD. Zambrano et al. showed that in mouse primary neuronal cultures, 50 µM ACV prevented the neuronal death and neurite disruption induced by HSV1 [8]. Lukiw et al. investigated the effect of ACV on miRNA146a, an miRNA implicated as a negative regulator of the innate immune system in AD and several other diseases, and found that 30 µM ACV significantly countered the increase in level of the miRNA caused by HSV1 [28]. These results support our findings in showing that an antiviral agent can reduce or eliminate the adverse effects of HSV1 on cells, in particular those that resemble the changes seen in AD, and hence support our contention that antiviral agents should be used for treating AD. However, we should stress, as we did in a recent review [29], that there are obvious limitations in comparing data on lytic infection of Vero cells with the changes seen in long-term neurodegenerative disease, just as there are limitations in deducing what changes occur in brain in early stage AD (few of which, obviously, are known) from the changes seen *post mortem.*

As well as the data supporting the role of HSV1 in AD, other researchers have argued against its role in the disease. One argument proffered is that HSV1 is too common to be involved. However, HSV1 infection is common only in the peripheral nervous system: at least 80% of the population have been infected with HSV1 by age 60 (previously, primary infection usually occurred in early infancy but with increasing socio-economic levels, it often now occurs at a later age). In contrast in the brain, it is usually absent in younger people: using solution PCR, we detected HSV1 DNA in many elderly brains but only in very few younger brains [30], and these results were substantiated by *in situ* PCR [14] and a totally different method, namely, detection of intrathecal antibodies in CSF from old people but not in CSF from infants [31]. Subsequently, the presence of HSV1 in the human brain was confirmed by several other groups (reviewed in [32]), using solution PCR. However, two studies found HSV1 DNA only very rarely in human brains [33], [34] and we have discussed the possible reasons for this [35], [36].

Another argument, against the role of HSV1 in AD is the lack of confirmatory studies showing that the combination of HSV1 in brain and possession of an APOE-ε4 allele is a strong risk factor for AD. In fact there is one study that showed consistent results [4] and a second that found a trend towards association [37]. No other relevant studies have been published. Also, in support of the HSV1-APOE connection are the studies showing that APOE-ε4 confers a strong risk of herpes labialis, a disorder of the peripheral nervous system known to be caused (usually) by HSV1 [3], [38] and also the studies showing the greater potential for HSV1 damage in infected APOE-ε4-transgenic mice compared to infected APOE-ε3 mice (reviewed in [32]).

A further argument against the role of HSV1 in AD is that Aβ and P-tau accumulation are not specific to HSV1. Indeed, other pathogens can produce AD-like changes – for example HIV causes Aβ and P-tau formation [39]–[41], and measles virus causes neurofibrillary tangles formation [42], but currently only HSV1, unlike these other viruses, has been detected in normal or AD brains, and so is the only candidate agent.

As well as undergoing a productive infection, HSV1 can undergo a latent infection in which no viral particles are produced and HSV1 gene expression is limited. However, stimuli such as stress and immunosuppression can reactivate the virus, leading to a productive infection. We have suggested that in later life the virus spreads to and becomes latent in the brain, and that during events such as immunosuppression, head trauma and peripheral infections, the virus reactivates and causes damage, including Aβ and P-tau formation. After each reactivation event, we suggest that HSV1 would spread (via intraneuronal pathways) and would thus become latent in additional cells. Repeated reactivation would lead to further spread and cumulative damage, which would develop into AD in those who possess an APOE-ε4 allele. Our concept of repeated reactivation is supported by the very recent finding that in infected mice, stimuli that induce HSV1 reactivation in the peripheral nervous system can cause it also in the brain, and that although virus production is limited to small regions, periodic reactivation induces areas of “focal reactive changes” [43]. Further, a repeated occurrence, rather than a single acute event, meets the requirement for a slow chronic disease.

Stopping spread of HSV1 might therefore slow the progression of AD. Although our results show that the antiviral agents we used did not stop Aβ accumulation completely, there was a statistically significant decrease. This decrease presumably reflects the inhibition of virus spread, which occurs as a consequence of the antiviral agents' effect on viral DNA replication, i.e., stopping viral DNA replication prevents complete virions from being formed and therefore stops the virus from spreading to additional cells. Therefore, our results suggest that antiviral agents, by preventing HSV1 spread as well as its replication, would provide effective treatment for the disease.

Our results suggest that ACV or PCV would be more suitable that FOS for use in AD since FOS was less effective at inhibiting HSV1 replication (consistent with earlier studies with this agent) and P-tau formation. A major consideration in the choice of an antiviral agent would be its ability to prevent or reduce HSV1 reactivation, as well as to reduce production of Aβ and P-tau. On that basis, PCV might provide a better alternative as some studies suggest that it can reduce the frequency or extent of HSV1 reactivation [44]-[46]. Also, although the differences in affinity of ACV and PCV for TK and the viral DNA polymerase, as mentioned above, probably balance each other, the intracellular half-life of the PCV triphosphate in infected cells is about 14 times that of ACV [26], suggesting that for clinical use, less frequent dosage might be needed.

Usage of an anti-herpetic such as ACV, or its biodrug valacyclovir (VCV), which has better oral bioavailability, to treat AD could prevent direct viral damage and, indirectly, HSV1-induced inflammation), irrespective of whether or not Aβ is involved. Clearly it would be preferable to start such treatment at as early a stage of the disease as possible when it is suspected in subjects with mild cognitive impairment. Another issue to consider is how effectively ACV would get to the brain in AD. However, this appears not be problematic since in people with normal blood-CSF barrier function, including those who have recovered from HSE, ACV (derived from VCV) has been shown to be present in CSF at the inhibitory level for HSV1 [47], [48].

The most usual target for current therapy is the elimination of Aβ production. However, if Aβ is in fact formed as an innate immune response but presumably – as we have suggested [32] – over-produced eventually, with consequent harmful effects, then its elimination might well be counter-productive. We therefore stress that antiviral agents would have an advantage in not eliminating Aβ but instead reducing it to a level closer to its normal very low value, which is approximately 10% of the HSV1-infected value. This could perhaps be achieved by using either another antiviral with a different mode of action that is more efficient than ACV or a combination of two antiviral agents operating via different mechanisms. Further, we stress that antivirals used in the current study are very safe and unlike other types of therapy, would not target any substance normally produced by the cell.

## Methods

### Viruses, cells and antiviral agents

We used HSV1 strain SC16, which was prepared as previously described [49], and HSV1 strain 17. Four derivatives of strain 17 were also used. Recombinant *in*1404 has an 8 base pair (bp) insertion in a HpaI site within the coding region of gE such that a termination codon was generated after codon 124 of the gE coding region. The mutant is predicted to synthisize a severely truncated protein, and cells infected with *in*1404 fail to bind rabbit IgG, indicatin that functional gE is not present [24]. Recombinant *ts*K/*lacZ* has a *ts* mutation in the coding sequences of ICP4 [23], [50], and in addition the virus used here has an insertion of the *E.coli lacZ* coding sequences controlled by the human cytomegalovirus major immediate early promoter (MIEP and simian virus 40 polyadenylation sequences inserted at the thymidine kinase (TK) locus). Insertion of the MIEP-lacZ cassette was achieved by co-transfection of ScaI-cleaved pMJ101 with *ts*K DNA, using methods described previously [51]. Plasmid pMJ101 consists of the MIEP-*lacZ* cassette inserted at SacI site within the TK coding region. ACV-resistant isolates were plaque purified and screened by Southern hybridization for disruption of a 2.4 kilobase pair EcoRI fragment that encompasses the TK coding sequences [51]. The recombinant *in*1863 was produced by co-transfection of ScaI-cleaved pMJ101 with DNA of 1814R, anHSV1, strain 17, isolate that is analogous to WT virus [52], using the methods described above for the construction of *ts*K/*lacZ*.

Recombinant *in*1374 has the ts mutation in ICP4, a deletion that inactivates the IE protein ICP0, an insertion that prevents stimulation of IE transcription by the HSV1 virion protein VP16, and the MIEP-*lacZ* insertion at the TK locus [22]. During infection at 38.5°C, *ts*K/*lacZ* produces IE proteins and beta-galactosidase (β-gal) but does not synthesize early or late proteins, whereas *in*1374 enters cells but does not produce detectable amounts of HSV1-specified gene products or β-gal in most infected cells [22]. Insertions of MIEP-*lacZ* were confirmed by Southern hybridization. The presence of the ICP4 ts mutation in *ts*K/*lacZ* and *in*1374 was confirmed by comparison of titres at 32°C and 38.5°C. The titre of *in*1374 was determined on the human osteosarcoma line U2OS at 32°C, in the presence of 3 mM hexamethylene bisacetamide. Under these conditions, the mutations in ICP4, ICP0 and VP16 are complemented, as described previously [22]. The other strain 17-derived viruses were titrated on baby hamster kidney (BHK-21) cells. The properties of the strain 17-derived viruses are summarised in Table 1. Staining for the presence of β-gal, and counterstaining of cells, were carried out as described previously [51].

**Table 1:** Properties of HSV1 strain 17-derived viruses.

Virus	Changes from WT HSV1	Properties	Reference
*in*1863	Insertion of MIEP-*lacZ* at TK locus	WT virus that expresses β-gal	
*in*1404	Insertion of 8 bp oligonucleotide in gE coding sequences	Does not express functional gE	[24]
*ts*K/*lacZ*	ts mutation in ICP4, insertion of MIEP-*lacZ* at TK locus	Overproduces IE proteins and expresses β-gal but does not synthesize early or late proteins at 38.5°C	[23], [50]
*in*1374	Mutations that inactivate VP16 transactivation function, ICP0 and ICP4, insertion of MIEP-*lacZ* at TK locus	Enters cells but synthesizes IE proteins and β-gal in only a small subset of infected cells	[22]

African Green Monkey kidney (Vero) cells (Health Protection Agency Culture Collections, Salisbury, UK) were cultured in Eagles modified essential medium containing non-essential amino acids, 10% foetal bovine serum, 4 mM glutamine, 10,000 U/ml penicillin and 10 mg/ml streptomycin. Cells were sub-cultured using trypsin-EDTA solution (T3924, Sigma-Aldrich).

ACV and FOS were purchased from Sigma-Aldrich, and PCV from VWR.

### Immunocytochemistry

For the experiments involving the recombinant viruses, Vero cells were seeded on to slides coated with 3-aminopropyltriethoxysilane, infected with HSV1 strain17 for 20 hours at an MOI of 5 and then fixed in phosphate-buffered saline (PBS) containing 4% formaldehyde and 10% acetic acid. For the experiments involving antiviral agents, 3-aminopropyltriethoxysilane-coated slides were seeded with 1.13×10^6^ Vero cells per slide and left overnight to settle. HSV1, at a MOI of 1, and ACV, PCV or FOS (at various doses) were then added. After 16 hours incubation, the cells were fixed in PBS containing 4% formaldehyde and 10% acetic acid.

Subsequently, slides were washed twice in Tris-buffered saline (TBS) for 5 minutes and then in 20% acetic acid to block endogenous alkaline phosphate activity. (For detection of Aβ1-42, slides had an initial treatment of 70% formic acid for 10 minutes and a three-minute wash in running tap water for antigen retrieval). The slides were then washed twice in TBS containing 0.025% Triton X and once in distilled water (each wash lasting five minutes), before being blocked for 60 minutes at room temperature in TBS containing 10% skimmed milk or TBS containing 10% goat serum and 1% bovine serum albumin. After two five-minute washes in TBS containing 0.025% Triton X, primary antibody was applied. Slides were incubated overnight at 4°C, washed in TBS containing 0.025% Triton X and biotinylated secondary antibody was added for 60 minutes. Subsequently, the slides were washed in TBS containing 0.025% Triton X and then treated with an avidin-alkaline phosphatase conjugate for 60 minutes before being washed in TBS. Substrate (BCIP/NBT) was then added until sufficient staining had developed. The primary antibodies used and their concentrations are shown in Table 2.

**Table 2:** Details of primary antibodies used for immunocytochemistry in this study.

Antigen detected	Host	Source	Product code	Dilution of primary	Incubation time	Secondary used
Amyloid β1-42	Rabbit	Abcam	ab10148	1 in 250	Overnight	Anti-Rabbit IgG
pS214	Rabbit	Abcam	ab10391	1 in 500	Overnight	Anti-Rabbit IgG
AT100	Mouse	Autogen Bioclear	90209	1 in 500	Overnight	Anti-Mouse IgG
HSV1	Rabbit	Abcam	ab9533	1 in 1000	Overnight	Anti-Rabbit IgG
ICP0	Mouse	In house	n/a	1 in 1000	Overnight	Anti-Mouse IgG
UL42	Mouse	In house	n/a	1 in 10,000	Overnight	Anti-Mouse IgG
gC	Mouse	In house	n/a	1 in 2000	Overnight	Anti-Mouse IgG
BACE1	Rabbit	Abcam	ab2077	1 in 250	Overnight	Anti-Rabbit IgG
Nicastrin	Rabbit	Abcam	ab24741	1 in 500	Overnight	Anti-Rabbit IgG
PKA	Rabbit	Abcam	ab26322	1 in 2000	Overnight	Anti-Rabbit IgG
GSK3β	Rabbit	Abcam	ab15314	1 in 100	10 mins	Anti-Rabbit IgG

For the nicastrin and BACE1 antibodies the blocking buffer used was TBS containing 10% goat serum and 1% bovine serum albumin. For the other antibodies 10% milk was used.

### Image J analysis

We quantified the ICC staining using the image analysis software Image J. This software assigns each pixel in the picture of the staining a value (between 0 and 255) which represents its brightness. Bright pixels have high values and dark pixels have low values. A range of pixel-brightness values is selected and any cells with a staining value lying within the range are regarded as positive; in the case of ICC the range is all pixels between a certain value and zero. This range is applied to all pictures of staining with the same antibody and the values produced represent the number of pixels showing positive staining.

We counted the number of cells in each field of view (using a phase-contrast image of the field) and used this value to determine the amount of ICC staining per cell. For each antibody we analysed three fields of view and used ANOVA to assess statistical significance. This method provides information about the amount of the target protein *in situ*, and so, unlike ELISA and Western blotting, does not depend on the efficiency of extraction of the protein, and also, rather than measuring the overall amount of target protein in or extracted from a presumed number of cells remaining after treatment, it can measure target protein in individual cells and the actual cell numbers. Further, this method overcomes the problems associated with any viral-induced changes in host cells (e.g., binding and aggregation of host proteins) that might affect their assay by techniques such as Western blotting and ELISA.

### Plaque reduction assay

24-well plates were seeded with 1.81×10^5^ Vero cells/well and the cells allowed to settle overnight. HSV1 was then added at a dose (0.0004 pfu/cell) that would result in the formation of approximately 80 plaques per well (i.e. a number that is easily countable and which would provide also an adequate statistical baseline). After incubation at 37°C for 1 hour, the HSV1-containing medium was replaced with medium containing 2% foetal bovine serum, 0.2% high viscosity carboxymethylcellulose and ACV, PCV or FOS (at various concentrations). After two days, cells were fixed with 10% formalin in PBS and stained with carbol fuchsin. Plaque numbers were counted and the dose of antiviral required to reduce infectivity to 50% (IC50) was calculated.

### HSV1 ELISA

96-well plates were seeded with 2.20×10^4^ Vero cells/well and the cells allowed to settle overnight. Cultures were mock infected or infected with HSV1 at a dose of 1 pfu/cell and incubated at 37°C for 16 hours. ACV, PCV and FOS (at various concentrations) were present throughout infection. Cells were fixed in acetone:methanol (1∶1) and then blocked with 10% skimmed milk. After 30 minutes of blocking, the plates were washed three times with 0.05 % Tween 20 in PBS and the primary antibody (anti-HSV1, abcam ab9533), diluted 1 in 8000 in blocking buffer, was applied for 90 minutes at 37°C. Subsequently, the plates were washed three times with 0.05 % Tween 20 in PBS and the secondary antibody (rabbit polyclonal to goat IgG HRP-conjugated (Abcam)), diluted 1 in 5000 in blocking buffer, was added. Following incubation at 37°C for 90 minutes and a further three washes with 0.05 % Tween 20 in PBS, peroxidase substrate (o-phenylenediamine dihydrochloride, Sigma), diluted in urea buffer, was added. After 30 minutes the reaction was stopped with 3 M hydrochloric acid and the absorbance at 492 nm was measured. IC50 values were calculated.
